# Work-Related Flow: The Development of a Theoretical Framework Based on the High Involvement HRM Practices With Mediating Role of Affective Commitment and Moderating Effect of Emotional Intelligence

**DOI:** 10.3389/fpsyg.2020.564444

**Published:** 2020-12-18

**Authors:** Xiaochen Wang, Shaheryar

**Affiliations:** School of Business Administration, Zhejiang Gongshang University, Hangzhou, China

**Keywords:** work-related flow, recognition, empowerment, information sharing, fair rewards, competence development, affective commitment, emotional intelligence

## Abstract

The long-term success of organizations is mainly attributable to employees’ psychological health. Organizations focusing on promoting and managing the flow (an optimal experience and optimal functioning state) may enhance employees’ well-being and performance to an optimum level. Surprisingly, the literature representing the role of HRM practices for their effect on work-related flow (i.e., intrinsic motivation, absorption, and work enjoyment) is very sparse. Accordingly, by drawing primarily on the job demands-resources model and HRM specific attribution theory, this paper develops a theoretical framework that unravels the effectiveness of specific organizational level High Involvement HRM (HIHRM) practices (i.e., recognition, empowerment, information sharing, fair rewards, and competence development) in activating the individual level work-related flow with beneficial effect and mediating role of affective commitment. In addition to highlighting the underlying mechanisms that may cause HIHRM practices to be regarded as resources and sometimes as demands, this paper especially proposes that these practices implemented with a focus to promote employee well-being are perceived as job resources and may positively influence affective commitment and flow, whereas these practices used as a demand to increase performance are perceived as job demands and may hinder affective commitment and flow. It is further significant to understand the possible moderating effects of emotional intelligence on the relationships among HIHRM practices, affective commitment, and flow. The paper augments the knowledge and understanding of the impact process of HIHRM practices, in particular how the HIHRM effect is sensed by the workers and thus, influences their succeeding job attitude and work experience. Finally, this work, as the first paper to link HIHRM practices with work-related flow, promotes the concept of positive psychology in the workplace.

## Practitioner Points

1. The paper presents a direction to live a meaningful and fulfilling work-life by consolidating the literature on specific HIHRM practices and linking these with work-related flow with the mediating role of affective commitment and moderating role of emotional intelligence.
2. The importance and positive and negative outcomes of flow have been discussed in the paper which provides practitioners with an awareness of the positive as well as negative aspects to emphasize on developing a flow-conducive workplace.
3. Additionally, the paper provides practitioners with insight into the levers they may use to promote and manage the flow within the workplace.

## Introduction

“Enjoyment appears at the boundary between boredom and anxiety, when the challenges are just balanced with the person’s capacity to act.” (Csikszentmihalyi, 1990, p. 52).

The “moment of highest happiness and fulfillment” is known as optimal experience and individual performance with complete efficiency in a task is considered as optimal functioning (Privette, 1983). In modern psychology, such a state that is related to peak experience and peak performance is known as “flow” which occurs when the balance between personal skills and task challenges is achieved by the individuals (Csikszentmihalyi, 1990; Jackson and Csikszentmihalyi, 1999). Flow is actually a figurative term to represent a state of mind of individuals when they are performing a task with such a deep involvement that they forget everything around them, they perform it with enjoyment and great efficiency (Csikszentmihalyi, 1990; Bakker, 2005). Even if people do not know the name of flow, they may have experienced it at one time or another. Chess, online games, and sports (e.g., football, hockey) are the most relevant examples of experiencing flow in which the attention is completely focused on the activity with feelings of amusement and high intrinsic motivation, and time flies. Such experience gives individuals happiness, makes them cognitively efficient, and hence they perform with their maximum capability (Csikszentmihalyi, 1990; Moneta and Csikszentmihalyi, 1996; Fullagar and Kelloway, 2009).

Flow experience in an organizational context is referred to as work-related flow (Bakker, 2005). Flow at work being a highly enjoyable and optimal functioning state protects employees from burnout symptoms like emotional exhaustion, cynicism, and inefficacy and has been found positively related to performance (Demerouti, 2006; Lavigne et al., 2012). Flow experience requires intense involvement and a keen interest in the activity (Csikszentmihalyi, 1990), it, therefore, positively predicts creativity and has a positive relationship with psychological capital (self-efficacy, hope, resilience, and optimism) (Zubair and Kamal, 2015). It may enhance the vigor and reduce the fatigue not only at work but also at home (Demerouti et al., 2012). While it has been recognized that flow has an important role in enhancing the richness of life, it is not free from negative consequences. Flow may have addictive properties as Csikszentmihalyi (2002) states that “the self becomes captive of a certain kind of order, and is then unwilling to cope with the ambiguities of life.” Also, the flow being based on challenges-skills balance requires individuals to look for more challenges, thus increasing their risk (Schüler and Nakamura, 2013). However, flow experience has a crucial role in the workplace and may generate the most significant and favorable outcomes for the organizations (Engeser and Rheinberg, 2008).

Nowadays, the ideal approach is to make employees happy and thus, productive to attain profitability (Oswald et al., 2015; Lan et al., 2017). However, different pressures at the workplace like target achievement, deadlines, task complication, job and task challenges seriously diminish employees’ well-being and make it difficult to keep employees psychologically healthy (Oswald, 2010; Böckerman et al., 2012). It seems critical for organizations to find out such mechanisms that may lead employees to work-related flow as individuals experiencing the flow find their work meaningful and enjoyable even when it is most difficult or challenging. Flow experience is undoubtedly a key construct of positive psychology and enhances employee contentment and performance to an optimum level (Csikszentmihalyi, 1990; Nakamura and Csikszentmihalyi, 2002). However, the studies related to predictors of flow experience in an organizational context are limited and they mainly focus only on situational elements (Bakker and Woerkom, 2017). To the best of our knowledge, the work-related flow has not been thoroughly explored from the HRM practices perspective. HRM practices have a pivotal role in influencing employees’ attitudes toward their work and increasingly gaining attention for organizations to attain a competitive edge (Chang et al., 2011; Lee J. et al., 2018). Additionally, while job characteristics and social exchange theories present some clarification of the link between HRM practices and individual-level outcomes, these relationships are still insufficiently studied (Guest, 2011; Shin et al., 2018). Specifically, the literature related to cross-level relationships is scarce (Jiang et al., 2013; Shin et al., 2018). There is an ample need to explore how certain organizational level HRM practices can be effective to enhance individual level work-related flow. Recognizing these gaps in flow experience and HRM literature, the paper theoretically explores not only the individual but also the collective influence of different HRM practices on the work-related flow.

Though the favorable effect of HRM practices is obvious, it is difficult to choose which practices should be used and combined to develop an effective HRM bundle. Paré and Tremblay (2007) and Yang (2012) recommend a multifaceted formation of five HRM practices: recognition, empowerment, information sharing, fair rewards, and competence development. These practices provide employees with the autonomy to make most of the decisions independently for their work, acknowledgment of employees’ efforts, impartial distribution of rewards, training and development, and information and feedback on how their actions affect workplace performance (Yang, 2012). The present paper focuses on these practices as we believe that these practices may connect and send signals to employees for the need of expected behavior that is valuable for the organization. Importantly, these practices are believed to be core HRM practices to trigger the high involvement of employees and therefore, called as High Involvement HRM (HIHRM) practices (Kilroy et al., 2017; Rubel et al., 2017). Flow experience also requires the highest involvement of individuals into the activity (Csikszentmihalyi, 1975, 1990) and thus, we expect that HIHRM practices may provide a platform to employees to experience the flow.

Researchers argue that HRM practices affect employee outcomes through an intervening mechanism (Guest, 2011). However, there is an uncertainty about this mechanism that is known as a “black box” in the field of HRM (Boxall et al., 2011; Rubel et al., 2017). So, it is necessary to further explore this “black box” and find out how HRM practices are delivered to employees. Accordingly, we posit job attitude (i.e., affective commitment) as the underlying mechanism through which employees’ perceived benefits from HIHRM practices transfer into work-related flow. There are a lot of variables that measure job attitudes but this paper emphasizes on affective commitment (AC) because it is related to emotional attachment that fits more appropriately in our model and with the aim of this paper. AC represents the likelihood of the formation of an emotional bond and elucidates that affectively committed employees are psychologically attached to the organization with feelings of affection, faithfulness, belongingness, passion, and pleasure (Jaros et al., 1993). It is the most effective type of job attitude as compared with a number of other related constructs as it significantly and positively influences employees’ behavior and performance (Camelo-Ordaz et al., 2011). Also, AC is believed to be correlated with a more productive and more positive affective state (Herrbach, 2006). It is reasonable to expect that AC may transform into a much more concentrated affective state (i.e., work-related flow), when it is influenced by HIHRM practices.

Emotions and emotional reactions may play a key role in the motivation mechanism involved in our proposed HIHRM practices-AC-work-related flow process relationship because HIHRM practices are more likely to be construed with emotions. Implementation of HIHRM practices may result in emotions like enthusiasm, pleasure, pressure, fear, anger, etc. For example, empowerment may develop a sense of responsibility that may come with emotions like excitement, nervousness, or uncertainty (Lan et al., 2017). Emotional exhaustion of employees decreases when they perceive that HRM practices have been implemented to support their well-being. In contrast, emotional exhaustion increases when employees feel that HRM practices have been introduced to reduce organizational costs (Shantz et al., 2016). As there is an involvement of emotions, we believe that emotional intelligence (EI) being an ability to recognize, comprehend, utilize, and manage emotions (Salovey and Mayer, 1997), may influence the underlying process of transmuting signals from HIHRM practices to work-related flow through AC. It is one of the important aspects of our paper because researchers emphasize that “emotions and emotional regulation in HRM” is a highly understudied area and requires comprehensive theory building (Ashkanasy et al., 2017).

This paper may make significant contributions. First, we examine the role of HIHRM practices (i.e., recognition, empowerment, information sharing, fair rewards, and competence development) in creating a balance between challenges and skills as it is believed to be a basic criterion to trigger the flow (Csikszentmihalyi, 1990; Bakker, 2005; Bricteux et al., 2016). Second, we propose that HIHRM practices implemented to enhance employee well-being are viewed as important job resources that may positively facilitate the work-related flow and AC. Third, we integrate HIHRM practices and AC to develop a theoretical model predicting the flow. The paper discusses the valuable effect and intervening role of AC. We presume that flow being an intense state of positive behavior is one of the meaningful outcomes of AC. The job demands-resources (JD-R) model, HRM specific attribution theory, and social exchange theory help in explaining the HIHRM practices-AC-work-related flow process relationship. Instead of drawing attention merely on the arguments related to positive versus negative aspects of HIHRM practices, the paper particularly focuses on the JD-R literature to view HIHRM practices as potential resources as well as potential demands. It clarifies the basic mechanisms that cause these practices to be seen as resources and sometimes as demands. HRM-specific attribution theory provides support to understand how specific attributions are formed about HIHRM practices in different situations that may result in a certain attitude and behavior. In addition, we principally argue that organizations using HIHRM practices to boost employees’ well-being are perceived as job resources and may promote AC and work-related flow. On the contrary, these practices may create stress and hinder AC and work-related flow when used as a demand to enhance employees’ performance because in such a case these practices are viewed as job demands. The fourth and final contribution of the paper is the identification of the contextual variable (i.e., EI). We expect that EI may affect the relationships between HIHRM practices and AC and further between AC and flow in a way that these relationships may become stronger. Moreover, employees with high EI levels are more capable to transfer HIHRM practices into work-related flow through AC. The paper considering HIHRM practices as an organizational level construct explores its effect on the individual level outcomes namely AC and work-related flow. The manifestation of EI as a moderating mechanism also takes place at an individual level. It is anticipated that this theoretical work will serve as a guideline and provide an impetus for future empirical studies. The paper also aims to extend the importance of flow experience in an organizational context, and this is why, below, we start with a discussion of flow experience and further explicate how proposed variables connect with each other.

## Literature Review and Hypotheses

### Flow: An Optimal Experience and Optimal Functioning State

The theory of flow was developed on the basis of widespread research conducted on the artists and sports professionals (e.g., music composers, basketball players, chess players), particularly the activities which were intrinsically rewarding. As a result, the first model of flow was introduced. This model conceptualizes flow experience as a balance between challenges and skills and explains that flow occurs whenever challenges of the activity are met by the required personal skills. It clarifies that high challenges with low skills produce anxiety and high skills with low challenges result in boredom (Csikszentmihalyi, 1975, 1990).

**FIGURE 1:**
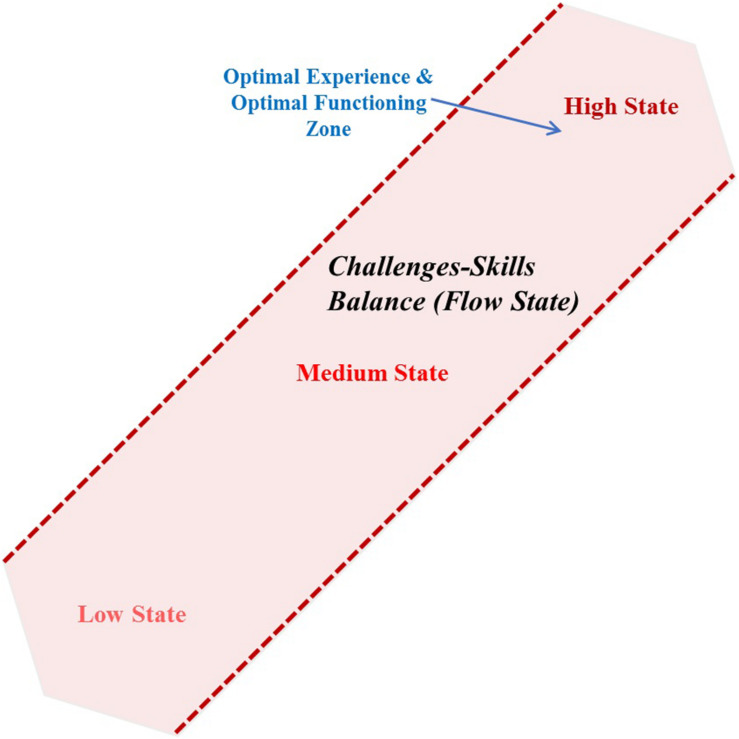
Flow channel.

Csikszentmihalyi and Nakamura (2010) argue that the ratio of challenges to skills should be exactly or very close to 50:50 to experience the flow as a minor mismatch may result in boredom or anxiety. Anxiety and boredom both are negative experiences. So, individuals will be motivated to escape such situations. If the individuals are in the boredom zone (having more skills but fewer challenges), they have a choice to increase their challenges to achieve the flow state (i.e., challenges-skills balance). Similarly, if the individuals are in the anxiety zone (having more challenges but fewer skills), they may either increase their skills or reduce their challenges to facilitate themselves to enter the flow state (Csikszentmihalyi, 1990). Defining the terms (i.e., challenge and skill) may enhance our understanding of the model. Challenge may be defined as a demanding situation having the highest chance for activity and growth, whereas skills are the competencies to deal with the challenge. Individuals in demanding situations perceive such situations as challenging but controllable with the help of their skills. Individuals believe that they may succeed to deal with a challenging situation by using their skills (Bricteux et al., 2016). For example, a skilled web designer may experience the flow while working on website illustration because his skills may meet the challenges of the task. Researchers reiterate that flow may occur when the challenges are met by needed skills for an activity (e.g., Csikszentmihalyi, 1975, 1990; Bakker, 2005). However, this model seems to be too simple. Flow may occur when the ratio of the challenges is equivalent to the ratio of skills for an activity, as shown in Figure 1. It will occur in all three cases (i.e., challenges and skills both are equal with their states as low, medium, and high). However, the intensity of the flow will be higher at the high state where the challenges and skills are balanced with high challenges and high skills than the low state where the challenges and skills are balanced with low challenges and low skills. Therefore, a new model suggested that flow may occur if these conditions are met: (1) challenges and skills are balanced, and (2) challenges and skills both are above average (Moneta, 2012).

Flow experience is intrinsically rewarding and comprises several other characteristics such as clear goals, the merger of action and awareness, high concentration, unambiguous feedback, and awareness of what an individual is doing but unconscious of surroundings (Csikszentmihalyi, 1975). It is related to meaningful moments of individuals’ lives because it is highly enjoyable and gives immense contentment and hence known as an optimal experience (Csikszentmihalyi, 1990). Individuals experiencing flow are completely immersed in the activity with great pleasure and they perform it with maximum efficiency and ability, that is why psychologists refer to it as “the zone of optimal functioning” whereas sports persons usually call it “being in the zone” (Prapavessis and Grove, 1991; Hanin, 2000). As such, flow is not only an optimal experience but also related to optimal performance (Privette, 1981). Thus, flow experience may be appropriately defined as

“A sense that one’s skills are adequate to cope with the challenges at hand in a goal-directed, rule-bound action system that provides clear clues as to how one is performing. Concentration is so intense that there is no attention left over to think about anything irrelevant or to worry about problems. Self-consciousness disappears, and the sense of time becomes distorted. An activity that produces such experiences is so gratifying that people are willing to do it for its own sake, with little concern for what they will get out of it, even when it is difficult or dangerous” (Csikszentmihalyi, 1990, p. 71).

Flow experience applies to every type of activity whether it is work or leisure. It is important to mention here that individuals having adequate skills enjoy the moments and activities the most which are challenging (e.g., difficult workplace tasks). Individuals often experience flow during their work time as they may deal with challenges frequently during work rather than in leisure time (e.g., watching TV) which has no challenges (Csikszentmihalyi and LeFevre, 1989). Taking this aspect into account, Bakker (2005) related flow to occupational tasks only and named it as work-related flow. He simplified the theory of flow in an organizational context by identifying resemblances among several definitions and concepts presented by different researchers and clarified that flow is a combination of three components: absorption, work enjoyment, and intrinsic motivation. Bakker (2005) defines absorption as a complete involvement of individuals in the activity in which employees forget everything around them and time flies, work enjoyment as a feeling of happiness that leads the employees to make a very positive image of their work-life, and finally intrinsic motivation as an inherent pleasure that employees get by doing the activity. The total involvement (i.e., absorption) of individuals into the activity with pleasure and fun (i.e., work enjoyment) and motivation to perform the activity for its internal reward (i.e., intrinsic motivation) makes complete sense concerning the concept of flow experience, particularly in a work setting. Gildemeister (2019) has also compared work-related flow components with original characteristics of flow. He describes that absorption appears to be a combination of a high level of attentiveness, loss of self-consciousness, a sense of control, clear goals, time transformation, and action and awareness merger. Work enjoyment resembles unequivocal feedback and particularly challenges-skills balance in that when employees have the necessary skills and resources to meet the challenges of a task, their work may turn enjoyable for them. Lastly, intrinsic motivation is similar to the autotelic aspect which is used to describe individuals who are internally driven. Bakker (2005, 2008) also developed a measurement scale namely work-related flow inventory which has proved to be a valid measure. Even empirical analysis of a study suggests that work-related flow scale is a better measure of flow as compared with older measures of flow (e.g., Flow State Scale-II) (Rupayana, 2008). A lot of studies have also successfully used the construct of work-related flow to relate it with different positive outcomes like performance, creativity, and energy after work (e.g., Demerouti, 2006; Demerouti et al., 2012; Zubair and Kamal, 2015).

In spite of the numerous benefits of flow experience, researchers argue that it may not be necessarily good to develop a culture of flow. Individuals experiencing flow are so deeply involved in the activity that they tend to do it even if it is extremely dangerous (Csikszentmihalyi, 1990). Flow has been found positively associated with risk-taking behavior due to loss of self-awareness (Schüler and Nakamura, 2013). For example, a study on big wave surfing (an activity involving flow experience) has found that big wave surfers are unwilling to quit big wave surfing even after injuries and harmful effects on their health (Partington et al., 2009). Flow is regarded as addictive behavior and researchers have found that it has a positive relationship with problematic internet use (i.e., excessive use of online gaming, social media, online searching, and buying, etc.) (Kim and Davis, 2009). Despite having a dark side, researchers recommend that individuals should seek flow because of its appealing and positive side (Schüler and Nakamura, 2013).

### HIHRM Practices Perspective

The high involvement model was presented by Lawler (1986), who proposed four organizational practices (i.e., information sharing, empowerment, rewards, and competence development) as core HRM practices for high involvement that may influence employees’ job-related attitudes. This model is regarded as a backbone for contemporary strategic HRM (McMahan et al., 1998). Recognition is one of the underlying dimensions of rewards, and rewards being a complicated process cannot be completely understood without recognition (Milkovich and Newman, 1998). As a consequence, studies suggested five HRM practices namely recognition, empowerment, information sharing, fair rewards, and competence development, and related these distinct practices to employees’ different positive work-related behaviors and attitudes (Appelbaum et al., 2000; Paré and Tremblay, 2007; Maden, 2015). These practices have been named most of the time as “high involvement” HRM practices (Paré and Tremblay, 2007; Maden, 2015; Kilroy et al., 2017). However, the common and basic purpose is to develop a system of organizational practices to provide organizational members with necessary skills, autonomy, and motivation (Wood, 1999).

#### HIHRM Practices and the Balance Between Challenges and Skills

In this global, competitive, and rapidly changing era, employees face different workplace demands or challenges routinely (Li and Li, 2016), and that is why they are continuously in a need to increase their skills. If the challenges are more than the skills for employees, they may face anxiety (Csikszentmihalyi, 1975); however, they can manage it by attaining the skills they require to deal with the challenges (Ceja and Navarro, 2012). Researchers consider competency as a key requirement of flow in a way that individuals are more likely to reach the state of flow when their skills match the challenges of the task (Csikszentmihalyi, 1975, 1990). It appears that employees with the help of HIHRM practices are expected to meet the challenges of their work as competence development practices are essentially used to increase the skills and abilities of employees through different developmental processes like mentoring, rotation, training and development, etc. (Paré and Tremblay, 2007; Maden, 2015). Empowerment provides challenges and satisfaction to employees at the same time (Lovelace et al., 2007). Information sharing practices may motivate employees to fully involve in their work (Rana, 2015). Similarly, fair rewards practices may inspire employees to work with full struggle and energies (Redmond, 2013). Moreover, the conformity preferences of employees help recognition practices to shape performance (i.e., low-performing employees increase efforts to better conform to high performing employees receiving recognition and similar benefits) (Bradler et al., 2016). It signifies that low skill employees are likely to make efforts to increase their skills to better conform to high skill employees who are empowered, provided with chances for competence development, kept well informed about organizational matters, recognized, or fairly rewarded for their skills and abilities. As such, HIHRM practices positively predict employees and firm performance by motivating employees not only to put extra effort because of increased control on their work but also to perform efficiently with improved skills and competencies (Pfeffer and Veiga, 1999). Thus, HIHRM practices (i.e., recognition, empowerment, information sharing, fair rewards, and competence development) may motivate and eventually furnish workers with all the skills that are essential to meet even high challenges of their jobs (Rubel et al., 2017).

The achievement of challenges-skills balance through HIHRM practices is also predictable when we see it through the lens of JD-R model. Researchers define job demands as “physical, psychological, social, or organizational aspects of the job that require sustained physical and/or psychological (cognitive and emotional) effort or skills and are therefore associated with certain physiological and/or psychological costs” (Bakker and Demerouti, 2007, p. 312). Job resources have been defined as physical, emotional, social, or organizational characteristics of the job that help to accomplish work-related objectives, minimize job demands or increase personal skills and competences (Conway et al., 2016). The JD-R model has two underlying processes consisting of health impairment and motivational processes. In the health impairment process, employees’ personal resources exhaust due to high-performance demands and become a source to deplete energy, which eventually may result in high levels of stress. The second process posits that employees’ motivation enhances due to job resources and leads toward a high engagement level (Bakker and Demerouti, 2007). Researchers view HIHRM practices (e.g., empowerment) as job resources to promote the employee well-being by providing autonomy, but they argue that these practices may also be perceived as extra work (i.e., job demands) when assigned against the employee expectations and may generate work stress (Lee A. et al., 2018). Similarly, there are several studies which emphasize on the positive impact of information sharing practices considering these as an important job resource for employees (e.g., Ahmad and Schroeder, 2003; Park, 2012, 2017), whereas some studies clarify that information sharing may also be viewed as job demand when it is overloaded (i.e., unnecessarily and irregularly shared with employees) and report that it is highly stressful (Klausegger et al., 2007). Besides, the key aspect is the organizational motive to implement HIHRM practices. We assume that HIHRM practices are viewed as job resources when organizations use these practices purely for employee development and as job demands when implemented as a demand to enhance employee performance. Consistent with the JD-R model, it is logical to suggest that HIHRM practices being potential job resources may help employees to meet the high demands of their jobs and hence may create challenges-skills balance. On the other hand, such practices may lead to stress and hinder flow when viewed as job demands.

#### HIHRM Practices and Work-Related Flow

**Recognition** may be precisely defined as a judgment and constructive response to an individual not only for his performance but also for his commitment and devotion (Brun and Dugas, 2008). It may be categorized as praise, written recognition, the employee of the month/year, representing company outside, public recognition, feedback, study assistance, conference attendance, presenting a gift, or even saying a simple “thank you” (Cacioppe, 1999; Schaetzle, 2015). By recognizing the efforts of employees, organizations can boost their morale and absorption in the work resulting in productivity and hence organizations may achieve profitability (Novak, 2016). Recognition practices make the work of the employees meaningful, build their identity, and positively enhance their well-being (Grawitch et al., 2006). Particularly, recognition practices enhance positive psychological functioning (i.e., enjoyment, resilience, optimism, autonomy, creativity, etc.) (Merino and Privado, 2015) and induce positive mood and pleasure implying that employees feel work enjoyment when they are appropriately recognized for their work and efforts (Argyle, 1997). Recognition of the work and accomplishments of individuals gives them a feeling of great pleasure which ultimately increases their internal motivation. Researchers and scholars, therefore, have always considered recognition practices as an important job resource to increase the intrinsic motivation (e.g., Deci and Ryan, 1980; Hansen et al., 2002; Fall, 2014). HRM-specific attribution theory (i.e., distinctiveness, consistency, and consensus) (Bowen and Ostroff, 2004) has a pivotal role in relation to HRM practices. HRM practices are believed to send signals and messages for which employees develop attributions about what is expected, required, and rewarded. HRM practices are delivered through the HR process that plays an important role to remove any ambiguity in delivery. A strong HRM system has an efficient HR process that makes HRM practices distinctive and consistent and helps developing agreement and shared perceptions among employees (i.e., strong climate) about HRM practices. Such a system has the potential to affect organizational effectiveness by efficiently conveying to employees what is expected and rewarded and motivating them to adopt desired attitudes and behaviors (Bowen and Ostroff, 2004; Ostroff and Bowen, 2016). Thus, organizations that have a strong HRM system may help employees in developing consensus, accurate attributions, and shared perceptions about what behavioral outcomes are expected and valued related to recognition practices. Resultantly, recognition practices may trigger employees’ motivation to put efforts to attain the desired result (e.g., work-related flow).

Though the positive effect of recognition is certain, Gimbel (2015) and Saunderson (2016) argue that “too much” recognition can be bad for employees. In particular, employees should not be appreciated for such a thing that is already expected. Unnecessary praise may develop a thought of employees that there is no scope for further improvement. Employees may also view “too much” recognition from a different angle (i.e., they are required to increase their performance). Employees who observe that organizations are recognizing them beyond their job performance (i.e., overrating) may feel a moral obligation and also pressure to increase their work-related efforts to match their performance with such recognition. In such a case, there are ample chances that employees may consider “too much” recognition as job demand that may create stress. Similarly, inadequate recognition (e.g., lack of appreciation) for a job well-done is one of the biggest sources of stress as employees feel underrated (Bhui et al., 2016). HRM-specific attribution theory clarifies that accurate attributions are only formed when there is consensus among employees in that the employees perceive HRM practices as fair with respect to three dimensions of organizational justice: distributive, interactional, and procedural (Bowen and Ostroff, 2004). Therefore, employees may not develop appropriate attributions and perform according to intended expectations in case of “too much” or lack of recognition by misinterpreting or considering these practices as unintelligible and particularly unfair. So, the ideal approach is to create an organizational environment where recognition practices are fair, well designed, and properly implemented. Such an environment automatically triggers the key motivational drivers of employees and they become eager to invest personal resources and energies (i.e., absorption) in their work with happiness (Schaetzle, 2015). Accordingly, it is predictable that employees who are regularly and fairly recognized for their efforts in an organization having a strong HRM system may consider such practices as job resources and may positively influence absorption, work enjoyment, and intrinsic motivation (i.e., work-related flow).

It is also important to understand that employees make efforts to know the organizational motives for using any of HRM practices. These practices may also be viewed from a different perspective (e.g., the organization requires more work efforts). It happens when employees view HRM practices as being implemented by the organization to enhance performance in order to gain a competitive edge instead of focusing on employee well-being (Jensen et al., 2013). This is the case when employees perceive such practices as job demands rather than job resources and their stress level increases and commitment level decreases as employees feel that organization is only interested in organizational profitability instead of employee development (Van De Voorde and Beijer, 2015). In line with the JD-R model, we may propose that employees consider recognition practices as job resources when organizations implement these practices to enhance employee well-being and may get the motivation to trigger the work-related flow, while employees viewing these practices as job demands (i.e., implemented by organizations to enhance performance) may feel stress and may not achieve the work-related flow state. So,

**Hypothesis 1(a).** Recognition practices may positively facilitate work-related flow when viewed as job resources, while may hinder it when viewed as job demands.

**Empowerment** provides an opportunity to employees to perform several roles and tasks, and that is why they have a larger effect at work along with increased autonomy (Paré and Tremblay, 2007). Job autonomy is believed to be an important job resource when it is provided as per employees’ expectations (Lee A. et al., 2018). The studies using the JD-R model also report that job autonomy is an important factor to promote flow (Bakker, 2005; Peters et al., 2014). Rana (2015) argues that autonomy may enhance the meaning of work for employees as they may exercise influence with a feeling of control and ownership of the work and valuable position in the workplace. Essentially, when employees feel that they have the freedom to schedule their tasks and have the power to decide that which technique to use to complete their tasks, their level of positive affect and motivation is increased which further leads them toward high levels of work immersion and pleasure (Fullagar and Kelloway, 2009). Rana (2015) also suggests that managers should enhance employees’ participation in solving work-related problems and use open communication showing high concern for employees to increase their engagement level. It is important to highlight that leaders who develop good relationships with employees and empower them may make their work extra challenging (requiring absorption) and extra satisfying that may lead employees toward motivation and eventually toward work enjoyment (Lovelace et al., 2007; Lee A. et al., 2018). A study in this regard also confirms that employees who are empowered, facilitated by teleworking, and receive leadership and collegial supports feel more flow at work (Peters et al., 2014).

However, researchers argue that empowerment is not necessarily always good because extra work and responsibilities may also be viewed as job demands by employees and may increase job stress. Specifically, employees should be empowered by keeping their expectations into consideration that how much additional work, autonomy, and decision making responsibilities they are willing and capable to handle, otherwise, they perceive empowerment practices as a burden that may create stress. The role of leadership is also crucial in empowering employees. Employees usually view empowerment practices as a sign that the leader has faith in them and providing them with opportunities for improvement and growth. However, employees through some evidence may also view such practices as an act of leader to avoid making difficult decisions. In such a case, employees become upset, consider empowerment as job demand, and feel stress. Resultantly, employees are unlikely to experience the flow and also may not perform well. Therefore, leaders should avoid putting too much burden on employees and create a trusting environment while empowering them (Lee A. et al., 2018; Wong and Giessner, 2018).

Individuals use internal and external attributions while looking for the answer to the question that why they or others behave in a certain way. For example, if an employee is empowered, he can accredit this to an internal attribution (the organization is interested to enhance his well-being) or an external attribution (the organization is complying with union requirements). Nishii et al. (2008) investigated this idea and found that employees’ attributions that HRM practices have been implemented to enhance their well-being are positively related to employee’s attitudes (i.e., commitment and satisfaction), whereas employees’ attributions that HRM practices have been designed to exploit employees are negatively related to employees’ attitudes. Thus, employees are expected to achieve work-related flow state by making positive attributions about empowerment practices particularly when they perceive that the organization is providing them an opportunity to improve and enhance their performance. Contrariwise, employees in negative scenarios (i.e., empowering employees against their expectations and in a distrustful environment) may ascribe empowerment practices to attributions that organizations have implemented these practices to take advantage of them; therefore, they may feel job strain and perform low and unable to experience the flow. So, the primary aim to implement empowerment or any other practices should be to promote employee well-being instead of focusing on any other organizational motive like requiring more work efforts from employees. Studies have clarified that the organizational motive other than the employee well-being while implementing HRM practices is viewed as job demand which increases emotional exhaustion (Shantz et al., 2016). Taking into consideration the JD-R model, we may hypothesize that,

**Hypothesis 1(b).** Empowerment practices may positively facilitate work-related flow when viewed as job resources, while may hinder it when viewed as job demands.

**Information sharing** is a practice of providing accurate information related to business results, goals, policies, changes, quality, and customer feedback to all the organizational members (Wood and Wall, 2007; Park, 2017). There is a consensus that information sharing is essential for organizations as it may generate positive outcomes. Park (2012, 2017) has found that employees usually regulate their behavior through the information provided to them. Information sharing increases the cognitive feature of work engagement by helping employees to find the connection between their actions and firm performance. Employees completely absorb themselves in their work when they are able to see the direct link between their actions and organizational outcomes through information sharing practices (Rana, 2015). Information sharing also conveys a message to employees that the company or firm trusts them, eventually promoting their involvement in the work (Paré and Tremblay, 2007). Importantly, there is ample evidence that trust is an important predictor of happiness (Bălţătescu, 2009; Jasielska, 2018) indicating that employees may eventually feel work enjoyment when they get adequate information through information sharing practices. Information sharing related to an organization’s financial position, ups and downs, and goals is very important as it generates feelings in employees that the organization has faith in their loyalty, skills, and judgment (Yang, 2012). These feelings help in developing a perception of employees that their contribution is valued by the organization toward the accomplishment of organizational goals. Such perception of employees is referred to as perceived organizational support. Employees regulate their behavior due to perceived organizational support and tend to put extra efforts to attain organizational goals (Park, 2017). It is evident from the literature that such efforts may result in organizational directed positive behavior of employees (Jiao et al., 2011). In fact, organizations enforcing HRM practices and consistently communicating with employees clarify the expected behavioral outcomes. Consistent information sharing and communication provide support to develop attributions and foster consensus among employees about the environment, hence employees are better able to understand HRM practices and may produce desired outcomes (Ostroff and Bowen, 2016). Park (2017) has also found that employees when feel that they have enough information about the company’s objectives, goals, and plans, they transform their behaviors to attain better performance. In short, organizations by distributing sufficient information to employees develop positive employee attributions about information sharing practices and motivate them to trigger absorption in their work with a feeling of enjoyment. Therefore, we believe that information sharing practices may create such contexts that may promote work-related flow.

Nonetheless, information sharing may also generate negative outcomes if it is overloaded. Information overload simply means extra and irrelevant information shared with employees through multiple sources in the organization which is difficult to understand and manage. Information overload occurs when the amount of information exceeds the individual’s capacity to process it. Resultantly, when information is overloaded, it is unlikely that the individual may work efficiently (Oldroyd and Morris, 2012). Studies have also confirmed that information overload being a job demand is highly stressful and has a negative relationship with the fulfillment of job responsibilities (Klausegger et al., 2007). Similarly, Lack of information sharing may enhance disputes and tensions which may badly affect the innovative behaviors (Vellecco and Mancino, 2010). Lack of information sharing results in stress and inefficiency as employees spend a lot of time searching for information and knowledge that is required to perform a task successfully (Robinson, 2018; Rosén, 2020). Applying HRM-specific attribution theory, we may deduce that if information sharing is overloaded or deficient in organizations, employees may develop negative attributions about information sharing practices and perform low by feeling stress. Such an environment may not be suitable for promoting work-related flow.

In the light of the above literature, earlier arguments, and JD-R model, we may propose that an efficient information sharing system (i.e., sharing sufficient but only necessary and relevant information through a well-organized setup) focusing on employee well-being is perceived as a job resource in organizations that may facilitate employees to absorb in their work with happiness and motivation that theoretically may result in work-related flow. Whereas, an information-sharing system that focuses to increase employee performance or represent information overload is regarded as job demand and may create stress and obstruct flow. Thus,

**Hypothesis 1(c).** Information sharing practices may positively facilitate work-related flow when viewed as job resources, while may hinder it when viewed as job demands.

**Fair rewards** may be defined as a practice of impartiality regarding different job results, which include performance appraisals, compensation, and job tasks, etc. It is related to individual perception of whether the allocation of resources, opportunities, and rewards is fair in the organization (Paré and Tremblay, 2007). Redmond (2013) explains that fair rewards are related to distributive justice. Distributive justice being based on the equity theory (Adams, 1963) endorses that the perception of fair rewards leads employees to adjust their work quantity and quality. As per equity theory, individuals compare their input (e.g., education, efforts, etc.) with their output ratio (e.g., salary, benefits, etc.). Kahn (1990) argues that rewards are given to individuals for the struggle and efforts they invest in their work and hence may influence meaningfulness. Similarly, Saks (2006) asserts that employees become willing to absorb in their work when they have an expectation of fair return against their investment. It implies that employees’ work should be designed in such a way that it may give them a sense of getting adequate return (i.e., compensation, rewards, and recognition) on their investment which may ultimately increase their involvement in the work.

Different rewards are perceived differently by employees concerning their motivation. Eisenberger and Shanock (2003) have found that rewards for novel performance enhance intrinsic motivation. Another study has revealed that extrinsic rewards strongly affect employee preferences, choices, and performance (Manolopoulos, 2008). Similarly, Markova and Ford (2011) have reported that non-monetary rewards significantly and positively predict intrinsic motivation. Rewards also positively predict work-related happiness. Several components of rewards have been found positively related to happiness such as meaningfulness at work, income, and opportunities for learning and personal development (Rego and Cunha, 2009; Oishi et al., 2011; Golparvar and Abedini, 2014). Fair rewards develop good feelings in employees because of the fair behavior of the organization and as a result, employees also tend to be fair in their task performance and respond with such behaviors that are valuable (Coyle-Shapiro et al., 2004). For example, employees may reciprocate with a high engagement level when they receive adequate rewards and recognition for their efforts (Maden, 2015). It implies that organizations by being fairer and transparent in their rewards practices motivate and influence employees to invest their efforts and energies in the work to repay. In fact, fair rewards may affect the decision making of employees to embrace the behavior defined in the competency model of the organization (Redmond, 2013). Thus, it can be concluded that organizations through their fair rewards practices convince employees to connect and absorb in their work and promote happiness and motivation or in other words, may instigate work-related flow.

Nevertheless, it is pertinent to mention that a large number of employees may be affected when the coverage of HRM practices is high. Resultantly, distinctiveness becomes high. High distinctiveness in regards to HRM practices may result in consensus which means that there will be more agreement among workers who will be receiving visible and consistent signals concerning the implementation of HRM practices. In the presence of consensus, more precise HR well-being attributions are anticipated that may increase employee commitment (Van De Voorde and Beijer, 2015). In line with this finding based on HRM-specific attribution theory, we argue that employees will perceive fair rewards practices in a more visible and consistent way in a highly distinctive situation and will develop positive attributions about fair rewards (i.e., distributive justice), resultantly they may perform as expected. On the other hand, in a low coverage scenario, fair rewards practices may not be applied to all the employees. Therefore, low coverage may result in inconsistency. Such inconsistency may convey a signal to employees that they are managed inconsistently in regards to fair rewards practices and they may feel partiality and irregularity in it. Hence, they may consider it an unfair reward system and unable to produce the desired outcome (e.g., work-related flow). Moreover, studies have confirmed that employees may also view compensation and benefits and other related HRM activities as the organizational desire to enhance performance (Koys, 1988). Summarizing all the above arguments, we may propose that fair rewards practices as being implemented by the organization to increase the performance with low coverage may be viewed as job demands and an unfair reward system that may create stress and hinder flow. Alternatively, high coverage of fair rewards practices with a focus to boost employee development may be perceived as job resources that may promote flow. Hence,

**Hypothesis 1(d).** Fair rewards practices may positively facilitate work-related flow when viewed as job resources, while may hinder it when viewed as job demands.

**Competence development** practices are used to enhance the productivity of individuals and convey a message to them that the firm is eager to invest in them. Such an investment may develop positive employees’ attributions and a moral commitment of employees to pay back through their efforts (Tzafrir, 2005). These practices are a major component of HIHRM practices, as it is critical that employees have the knowledge, skills, and abilities to consider and discuss options to make accurate decisions related to their work (Konrad, 2006). A study by Shuck et al. (2014) has revealed that cognitive, emotional, and behavioral engagement can be increased by the employees’ participation in human resource development practices like learning opportunities, leadership development initiatives, and mentoring programs. These practices are regarded as one of the important factors to increase motivation, interest in learning and exploring new things, performance, productivity, innovative capability, and to gain a competitive edge (Eilström and Kock, 2008; Muñoz Castellanos and Salinero Muñoz, 2011). Studies have also identified that organizations fostering learning and competence development promote employee health and well-being (Karasek and Theorell, 1990). Competences have a significant and positive relationship with intrinsic career success (job satisfaction) and extrinsic career success (Hennekam, 2016). Competences are also viewed as an important job resource to perform a complicated or challenging task successfully (Bergenhenegouwen et al., 1996). More importantly, competence development practices provide contentment, prepare employees to meet the high challenges of their job, and a possibility to attain a better fit with their desired self-images due to the disclosure of alternative roles (Rana, 2015). Therefore, these practices may play a key role in achieving the challenges-skills balance that ultimately may activate work-related flow.

In accordance with HRM-specific attribution theory, organizations should focus to develop a strong HRM system based on distinctiveness, consistency, and consensus with regard to HRM practices (e.g., competence development practices) as it may develop attributions and shared perceptions of employees (i.e., strong climate) “for a particular strategic focus” (e.g., improved performance) (Ostroff and Bowen, 2016). Thus, employees may easily understand and follow the guidelines of organizations regarding competence development practices in an environment having a strong HRM system. Organizations may not motivate employees to adopt competence development practices if they have ambiguity about the purpose of such practices. Ambiguity affects the comprehension and hampers the process of consensus and may not generate precise attributions about HRM practices (Bowen and Ostroff, 2004).

Competence development practices may also generate stress and hinder the employee skill development process. It is because competence development practices are mostly implemented due to competition pressure, clients’ demands for improved services, and better product quality or technological changes instead of employee well-being, hence represent job demands (Eilström and Kock, 2008). Employees may not consider such practices as job resources when they feel that these practices have been implemented with an aim to meet certain organizational demands. In such a case, it is most probable that employees may develop negative attributions that the organization is least interested in employee well-being and may not perform as intended. The organizational motive to implement any of HRM practices should be to promote employee well-being as it may reduce employees’ emotional exhaustion (Shantz et al., 2016). Studies emphasize that organizations should provide workers with resources in a trusting and helpful environment as it encourages proactive behaviors leading toward happy, skilled, and innovative staff. Specifically, if employees’ needs are considered with a chance of negotiation and adjustment to the priorities and choices of employees while implementing HRM practices, HRM can be certainly sustainable (Villajos et al., 2019).

As per JD-R model, it is reasonable to propose that employees consider competence development practices as job demands when they observe that these practices have been implemented to increase performance in order to meet internal or external demands. Consequently, employees may feel stress and may not be motivated to learn new skills, and hence it is unlikely that they may experience the flow. Contrariwise, employees view such practices as job resources when they feel that these practices have been implemented to support their well-being. In such a case, employees may get the motivation to put efforts to attain the necessary skills and competencies that may help them to meet job challenges and hence may positively influence the flow. Therefore,

**Hypothesis 1(e).** Competence development practices may positively facilitate work-related flow when viewed as job resources, while may hinder it when viewed as job demands.

**Concluding** all the above arguments, HIHRM practices collectively as a bundle being job resource may reform the job experience in such a manner that it becomes more motivating for the individuals, produces pleasure and enjoyment, increases focus and performance, and induces positive behavior, involvement, and engagement (i.e., vigor, dedication, and absorption) (Boxall et al., 2015; Rana, 2015; Huang et al., 2016; Gupta and Sharma, 2018). We expect that HIHRM practices collectively may significantly activate flow at work as these practices altogether may furnish employees with a high skill set to deal with high challenges, high absorption, high intrinsic motivation, and greater work enjoyment when viewed as job resources. Researchers have also stressed upon the collective role of HRM practices. They argue that different HRM practices may be bundled together to look for their differential effect on employee outcomes. In fact, such a bundle based approach delivers a significant insight that which set of practices may be used to obtain desired behavior (de Reuver et al., 2019). In a similar vein, Shin et al. (2018) have reported that HIHRM practices bundled together may significantly and positively induce intrinsic job motivation and further lead to creativity. Several other studies also provide evidence that HIHRM practices (i.e., recognition, empowerment, information sharing, fair rewards, and competence development) have a more positive and powerful influence when bundled together rather than using separately and these practices as a whole may result in numerous positive outcomes for employees (e.g., Rana, 2015; Kilroy et al., 2017; Rubel et al., 2017).

It is important to note that the implementation of HRM practices highly depends on the management’s beliefs and its idea about the value of its human and social capital (Macduffie, 1995). In line with the resource-based perspective, companies apply a quality enhancement HR strategy when they perceive employees as assets needed to produce premium quality goods. Companies invest in their employees for the long-term development of their skills. The focus of management in such companies is on the employees’ motivation so that they may work hard, and the management gives more importance to employees’ well-being than the company’s profit. On the other hand, when the management is more concerned about cost efficiency, it observes employees’ output and pays attention to manage employees to ensure their compliance with rules and regulations (Nishii et al., 2008). Accordingly, two connotations namely commitment-focused connotation and control-focused connotation have been suggested in the taxonomy of employee-oriented ideology. These connotations have been labeled as “employee well-being” and “exploiting employees” respectively. Employee well-being refers to employees’ attributions that HRM practices have been applied to promote employee well-being. Such attributions are positively related to employees’ attitudes (i.e., commitment and satisfaction). Whereas exploiting employees refers to employees’ attributions that HRM practices have been implemented to reduce cost and exploit employees. Such attributions are negatively related to employees’ attitudes (Nishii et al., 2008). These findings suggest that organizational intention to implement HRM practices plays a key role in the development of employees’ attributions. In this regard, studies have further clarified that demanding an increase in employee performance or conveying similar intentions through HRM practices may develop performance-focused HR attributions. In such an environment, employees consider HRM practices as job demands and feel stress that can be harmful for their health and may also result in decreased performance instead of any positive outcome (Hamid et al., 2015; Van De Voorde and Beijer, 2015; Sidhu et al., 2020). Conversely, organizations concentrating on employees’ well-being or sending such signals through HRM practices which signify that organization is interested in employee development may develop well-being-focused HR attributions. Such attributions enhance employees’ commitment and psychological health that may ultimately increase employees’ and organizational performance, hence mutually beneficial (Van De Voorde and Beijer, 2015; Bubonya et al., 2017; Guest, 2017). Accordingly, based on the JD-R model, we may propose that,

**Hypothesis 1 (f).** HIHRM practices (i.e., recognition, empowerment, information sharing, fair rewards, and competence development) collectively may significantly and positively facilitate work-related flow when viewed as job resource, while may hinder it when viewed as job demand.

#### HIHRM Practices and Affective Commitment

Although HIHRM practices have a positive relationship with flow experience, we expect that it is improbable that these practices may create a direct link with the flow. It is because flow experience is a state of high concentration and even total involvement in the activity (Csikszentmihalyi, 1990), and such a state does not seem to be activated by the straight effect of HIHRM practices. Researchers also assert that HRM practices affect employee outcomes through an intervening mechanism (e.g., Guest, 2011). Thus, we propose AC as a mediating mechanism through which the HIHRM effect is converted into work-related flow. AC is the core component of organizational commitment. It has been defined as “an emotional attachment toward the organization such that the strongly committed individual identifies with, is involved in, and enjoys the relationship with the organization” (Sanders et al., 2008). AC fits better in our research model as compared with other components of organizational commitment. AC is related to intrinsic motivation and hence more likely to be associated with a more positive attitude and behavior than continuance commitment which is related to feelings of pressure and normative commitment which is related to feelings of obligation (Meyer et al., 2002; Grant et al., 2008). Moreover, AC is also more strongly linked to organizational citizenship behavior, attendance, and performance as compared with continuance and normative commitments (Meyer et al., 2002). Studies considering AC as an outcome have reported its different predictors like perceived organizational support in terms of care for employees and valuing their contributions, perceived career support, and several organizational practices (Vandenberghe et al., 2004; Marescaux et al., 2013; Poon, 2013). Many studies aiming to explore the effects of AC relate it with different positive work-related outcomes (Ng and Feldman, 2011; Schmidt and Diestel, 2012; Rivkin et al., 2018).

After satisfaction of the fundamental financial necessities of life, our focus of attention turns to other elements and some of our basic needs which are more important for our work lives, particularly different HRM practices like recognition, empowerment, information sharing, fair rewards, and competence development, etc. Employees’ perception of HRM practices as being used to increase their well-being is a very important aspect. Studies provide evidence in this regard by presenting a positive relationship between employee HR well-being attributions and organizational commitment (Koys, 1988; Nishii et al., 2008). Another study has also revealed that individuals’ insight into the HRM system (i.e., distinctiveness, consistency, and consensus) positively predicts AC (Sanders et al., 2008). Moreover, the social exchange theory (Blau, 1964) provides ample support for interpreting the relationship between HIHRM practices and AC. This theory explains that organizations through some certain practices (e.g., HIHRM practices) convey a message to employees that they care for their individual needs and recognize their contributions (Eisenberger et al., 1986). The norm of reciprocity plays an important role in this theory as individuals in such an organization repay with their positive attitude and behavior (Takeuchi et al., 2007; Alfes et al., 2013). Essentially, an environment that is characterized by HIHRM practices (i.e., recognition, empowerment, information sharing, fair rewards, and competence development) in the form of job resources (i.e., focusing on employees’ well-being) develops a feeling in employees that they are fairly treated, appropriately rewarded, and valuable resources of the organization, and hence leads employees to behave favorably particularly in terms of their affective attachment to and identification with the organization (i.e., AC) (Paré and Tremblay, 2007; Yang, 2012; Maden, 2015; Rana, 2015). A job environment that offers resources builds a positive image of the organization in employees’ minds. Employees consider resources as organizational support and their comfort level increases. They feel emotionally satisfied and, as time passes, they develop an emotional attachment with the organization. Therefore, an increase in job resources may increase the overall AC (Rousseau and Aubé, 2010). Conversely, when employees feel that their well-being has not been taken into consideration in the implementation of a high-performance work system, they view it as a burden, extra work and demands, and their feelings of stress increase and feelings of commitment decrease (Van De Voorde and Beijer, 2015). Job demands deplete employees’ energetic resources and may result in exhaustion. Employees withdraw mentally while coping with such exhaustion and hence may not influence AC or their AC level decreases (Sharma and Dhar, 2016). So, in the light of social exchange theory and JD-R model, the role of HIHRM practices in influencing AC may be summarized as under:

**Hypothesis 2.** HIHRM practices (i.e., recognition, empowerment, information sharing, fair rewards, and competence development) may positively influence AC when viewed as job resource, while may hinder it when viewed as job demand.

### The Beneficial Effect and Intervening Role of Affective Commitment

As the model of flow relies on the balance between challenges and skills, it implies that flow is facilitated by the task that is challenging. Therefore, studies suggest that the investment of energetic resources by an individual is the psychological precondition of flow experience (Nakamura and Csikszentmihalyi, 2002; Rivkin et al., 2018). AC satisfies this precondition as employees with high AC levels are expected to put a great deal of effort to achieve the organizational goals because of strong faith in the values of the organization (Park and Rainey, 2007). Moreover, studies suggest that personal and task characteristics facilitate the flow (Nakamura and Csikszentmihalyi, 2002; Debus et al., 2014). AC is also one of such externally influenced characteristics that may facilitate flow experience. The reason is that employees low in AC may not devote their energetic resources in the work because they are more likely to do the minimum amount of work that is required by the rulebooks of the workplace. In comparison, employees with high AC levels are more likely to devote their energetic resources in the work because of their emotional attachment and as they are more predisposed to perform beyond the formal requirements of work (Rivkin et al., 2018), hence may achieve challenges-skills balance.

AC is a happiness-related construct associated with experiencing a positive affect that has meaningful outcomes for the individuals and organizations. Employees high in AC respond with more positive behavior (Meyer and Herscovitch, 2001; Herrbach, 2006; Fisher, 2010). Flow experience is also a happiness-related construct but it is an intense state of positive behavior with the highest intensity of involvement (Csikszentmihalyi, 1975, 1990). It is, therefore, logical to assume that employees who are influenced by HIHRM practices may pass through an affective state of commitment prior to experiencing the flow. Affectively committed employees feel more optimistic and more enthused, they experience low ego depletion, low need for recovery, high work engagement, and high subjective vitality. It is for this reason that AC positively predicts employees’ psychological health (Meyer and Maltin, 2010; Rivkin et al., 2018). A high level of employee commitment in organizations may result in fewer turnovers, generate a high sense of self-worth, self-esteem, feeling of being important, and lead toward increased teamwork (Stone, 2008). Affectively committed employees consider their work meaningful and they have a feeling that they have enough resources to complete their work (Shuck et al., 2011). Moreover, affectively committed employees are more pleased with their job and hence they are more motivated to devote their efforts and time in their work with full concentration. They are able to easily connect and involve in their work (Poon, 2013).

The characteristics of flow experience (i.e., a balance of challenges and skills, action and awareness merger, explicit goals, unequivocal feedback, loss of self-consciousness, a sense of control, attentiveness on the task, time transformation, and autotelic experience) reflect such a condition in which the persons are completely immersed in the task with pleasure and get their motivation by doing that particular task rather than from extrinsic rewards which may be related to the task (Bakker, 2005). Resultantly, the flow experience collectively reveals high levels of intrinsic motivation (Rivkin et al., 2018). Researchers argue that AC is also positively linked to high intrinsic motivation (Meyer et al., 2002; Grant et al., 2008). The conceptual emphasis on AC is congruous with social exchange theory, which evinces that the satisfaction of workers’ fundamental needs through practices like HIHRM practices (i.e., recognition, empowerment, information sharing, fair rewards, and competence development) fosters autonomous regulation and hence especially promotes AC. Compared to controlled regulation, that is essential when looking for extrinsic rewards; autonomous regulation suggests that tasks are chosen without restrictions and congruous with an individual’s core values. The essence of social exchange theory cannot be predetermined. Thus, it emphasizes the long-term and consistent exchange of resources (Yiğit, 2016). In this line of reasoning, we argue that if the organization continues to deliver HIHRM practices, employees’ AC level also continues to increase. At a certain level, such commitment is firmly reciprocally linked to high intrinsic motivation (Meyer and Maltin, 2010; Rivkin et al., 2018). As such, employees with high AC levels may completely absorb in their tasks with enjoyment and high intrinsic motivation (Meyer and Maltin, 2010; Poon, 2010, 2013), or in other words may activate work-related flow.

Finally, it is pretty logical to determine that individuals perceiving HIHRM practices as job resources may achieve high levels of AC. Further, individuals with high levels of AC may experience flow at work because they are expected to invest their energetic resources in the work (i.e., satisfying the balance between challenges and skills) and as they may become more involved and enjoy their work with high intrinsic motivation than uncommitted individuals. Taken together, these arguments signify that AC is the more proximal antecedent to work-related flow and hence suggest a model in which the effect of HIHRM practices on work-related flow is transmitted through AC.

**Hypothesis 3. (a)** High levels of AC may positively predict work-related flow and **(b)** the positive relationship between HIHRM practices (i.e., recognition, empowerment, information sharing, fair rewards, and competence development) and work-related flow may be mediated by AC.

### The Moderating Effect of Emotional Intelligence

EI is a skill to manage emotional affairs (Wong and Law, 2002). It has been precisely defined as a “competency in perceiving, understanding and regulating our own emotions and the emotions of others” (Davis and Nichols, 2016). EI may lead to success in an organization because people with a high EI level may appropriately use this capability for making decisions, developing, and strengthening their relationships with people (Cooper and Sawaf, 1998). It has been linked with different positive outcomes like job satisfaction and well-being and has also been found positively related to low levels of cynicism and burnout (Brunetto et al., 2012; Kaur et al., 2013). Ashforth and Humphrey (1995) argue that emotions are rooted in all the activities of employees. They also state that emotions and emotional reactions to daily events have effects on the behavior of employees. EI being an ability to use and manage emotions may wisely influence and control job attitudes and behaviors (Lazarus, 1991; Choi et al., 2011).

Researchers also assert that all HRM activities involve emotions. For instance, the unfair distribution of rewards may induce negative emotions whereas fair rewards may promote positive emotions in employees. Therefore, handling rewards and compensation-related matters requires an understanding of emotions (Ashkanasy et al., 2017). In previous sections, we have argued that HIHRM practices as being implemented to increase the performance are perceived as job demands whose prolonged exposure may result in burnout (Bakker et al., 2004). However, individuals with a high level of EI are more efficient in controlling stressful moods and managing impulses (Daniel et al., 2002). An empirical investigation has also revealed that EI diminishes the effects of negative emotions on burnout (Szczygiel and Mikolajczak, 2018). EI not only manages distressing situations, but also efficiently deals with anger and sadness (Mikolajczak et al., 2008). On the other hand, HIHRM practices as being implemented to support employee well-being are perceived as job resources resulting in positive emotions that may increase employee engagement level (Bakker and Demerouti, 2007). It is important to mention here that high levels of EI represent a state of high awareness of positive emotions. Therefore, employees with high EI levels are more careful and more responsive to positive appealing practices and experiences at work (Zeidner et al., 2004; Lan et al., 2017). In short, emotionally intelligent employees may mitigate any negative signals received through organizational performance-focused motive and efficiently construe the positive signals conveyed through organizational well-being-focused motive in regards to HIHRM practices. They are more likely to produce a positive outcome even in an undesirable environment. Magnano et al. (2017) also confirm that EI reduces the harmful effects of psycho-physical exhaustion on the organizational behavior, lessens the risk of behavioral disengagement while coping with stress, frustration, and difficulties, and enhances the performance. Thus, emotionally intelligent employees are expected to latch onto and appreciate the importance of HIHRM practices, and hence may respond with a positive attitude and behavior like AC irrespective of any organizational motive.

On the other hand, employees with low EI have insufficient emotional drive and competency to get benefit from positive relational resources such as HR systems in activities requiring continuous attention and emotional self-motivation (Durán et al., 2010; Mossholder et al., 2011; Lan et al., 2017). Moreover, employees with low EI levels are less attentive to the emotional details. Such employees also have low levels of understanding of how emotional information can lead to a specific work attitude (Salovey and Mayer, 1990; Lan et al., 2017). That being the case, employees with low EI are expected to respond less strongly to HIHRM practices, ultimately weakening the influence of HIHRM practices on AC.

**Hypothesis 4(a):** EI may moderate the positive relationship between HIHRM practices and AC in such a way that the relationship will be stronger in the presence of high levels of EI.

The high levels of AC may generate an emotional state of work enjoyment, absorption, and high intrinsic motivation (i.e., work-related flow), as mentioned earlier. To achieve such an emotional state, employees should have the ability to transform AC into a concentrated effort. EI may play a crucial role in it as employees with high levels of EI may use their emotions to facilitate their work (Wong and Law, 2002; Lan et al., 2017). It suggests that employees with high levels of EI have the skill to get the benefit and they are more probable to give value and appreciate the high levels of AC. Accordingly, they are more likely to transform AC into an ideal state of work that is total involvement in the work (absorption), with great pleasure (work enjoyment), and high intrinsic motivation, collectively named as work-related flow.

In contrast, individuals having a low level of EI have less control over their emotions and hence it is difficult for them to use their emotions in effective manners and they may also find it difficult to focus, learn, and control their feelings and actions (Goleman, 1996; Zhou and George, 2003). Thus, they are inefficient to appropriately appreciate the high levels of AC. Such individuals are less likely to enjoy, concentrate, and absorb in their work or in other words less likely to experience the flow at work (Lan et al., 2017). These arguments are summarized as follows:

**Hypothesis 4(b):** EI may moderate the positive relationship between AC and work-related flow in such a way that the relationship will be stronger in the presence of high levels of EI.

Putting all the previous discussions and arguments concerning the role of EI together, we may determine that the paper proposes a moderated mediation effect of EI in a way that EI will enhance the indirect influence of HIHRM on work-related flow through AC. This justification for the moderated mediation effect is in line with a study by Preacher et al. (2007). Researchers report that emotions are related to “affective events” in organizations. Affective events activate emotional reactions which consequently trigger “affect-driven behavior” (i.e., behavioral reactions) and job attitudes like commitment and/or job satisfaction. Such behavioral reactions and attitudes ultimately lead toward well-thought-out “judgment-driven behaviors.” So, from the HRM viewpoint, management of the behavior of employees is essential and EI plays a key role in it (Weiss and Cropanzano, 1996; Ashkanasy et al., 2017). It signifies that when employees will be in a better position to unravel and utilize the positive emotional information and resources involved in the process, their positive perception of HIHRM practices, associated AC, and their work-related flow experience will escalate. Thus, we assume that employees with high EI will be more skilled to transfer the HIHRM effect into work-related flow through AC. On the contrary, employees with low EI will be less likely to trigger the HIHRM effect to AC, and the state of AC is also difficult to be activated as work-related flow. So, AC in a low EI situation is less likely to play a significant role in linking the HIHRM practices with work-related flow. We, therefore, propose that,

**Hypothesis 4(c):** EI may moderate the strength of the mediated relationship between HIHRM and work-related flow through AC in such a way that the mediating role of AC will be stronger in the presence of high levels of EI.

**FIGURE 2:**
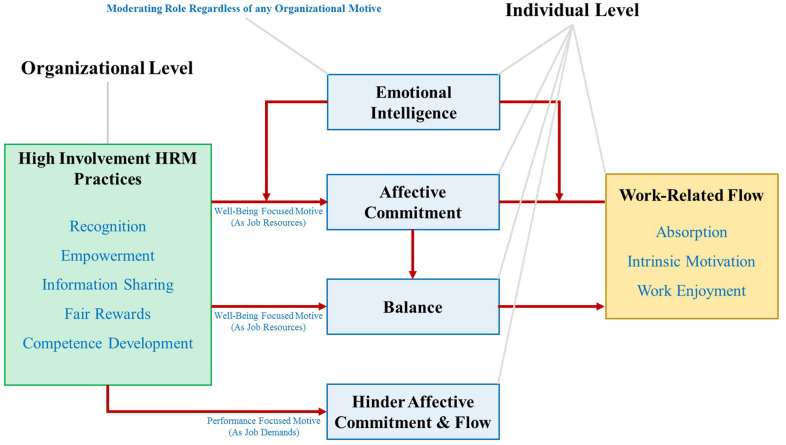
Theoretical framework.

The theoretical framework in relation to the above literature is presented in Figure 2.

## Discussion and Conclusion

This paper is the first to present a theoretical research model for the relationship of HIHRM practices with work-related flow. It augments the positive psychology at the workplace, especially the HRM and organizational psychology fields. It is of utmost importance that organizations should focus on improving employees’ psychological health as it has a crucial role to enhance performance and productivity (Bubonya et al., 2017). The present paper is an important theoretical contribution as it reveals that organizations using HIHRM practices (i.e., recognition, empowerment, information sharing, fair rewards, and competence development) to promote employees’ well-being may instigate work-related flow with the mediation of AC and moderation of EI. Flow being related to optimal experience and optimal functioning covers both aspects (i.e., positive mood and performance) (Csikszentmihalyi, 1990; Demerouti, 2006; Zubair and Kamal, 2015).

### Applicability of Framework

HIHRM practices model has been applied by researchers in more or less every sector of the economy like banks, health care, financial services, education, telecommunication and technology, etc. to predict different positive workplace outcomes (Maden, 2015; Kilroy et al., 2017; Rubel et al., 2017). Similarly, the construct of flow has also been successfully investigated in a variety of occupations like manufacturing, hotel, elderly care, accountancy, teaching, hospitals, lawyers, marketing, sales, travel agents, etc. (please refer to Nielsen and Cleal, 2010; Rodriguez-Sánchez et al., 2011; Colombo and Zito, 2014; Bricteux et al., 2016; Lan et al., 2017). Accordingly, the proposed research model is applicable in almost every work section, particularly where employees face work-related challenges on a routine basis. However, the influence of HIHRM practices on work-related flow is expected to be different in every sector of work as it mainly depends on the level of skills and challenges. HIHRM practices may instigate work-related flow at a lower state in organizations where challenges and skills are balanced with low challenges and low skills and at a higher state in organizations where both are balanced with high challenges and high skills (refer to Figure 1). For instance, HIHRM practices are likely to stimulate work-related flow at a higher state in the game development job as it is considered as most challenging job and at a lower state in the hairstylist job due to no or fewer challenges.

### Theoretical Implications

The paper supports the idea that HRM practices may send signals in either way: employees are perceived as valuable resources of the organization or they are expected to increase their performance (Kroon et al., 2009; Van De Voorde and Beijer, 2015). Consistent with the JD-R model (Bakker and Demerouti, 2007) and HRM specific attribution theory (Bowen and Ostroff, 2004), HIHRM practices (i.e., recognition, empowerment, information sharing, fair rewards, and competence development) seen as care and support for employees can be a signal and job resource which may positively predict high levels of AC and flow experience. On the other hand, these practices seen as a demand to increase the performance may create job strain and obstruct AC and flow. Researchers have emphasized that research with regard to HRM system strength requires extension as it is an under-investigated concept (Ostroff and Bowen, 2016). The present paper is an important step to extend the knowledge in this area particularly in terms of the identification of a bundle of specific HIHRM practices. First, we argued that HIHRM practices (perceived as job resources) may create challenges-skills balance and positively facilitate the flow. It supports the argument for making employees highly involved through HIHRM practices. This is due to the fact that flow experience is also a state of high involvement (Csikszentmihalyi, 1990). Second, HIHRM practices (perceived as job resources) are positively related to AC which further predicts work-related flow. As far as we know, this paper is the first to explore the underlying mechanism (i.e., AC) that determines the positive effect of HIHRM practices on work-related flow. The paper explains that AC that is influenced by HIHRM practices may motivate employees to invest their energetic resources, connect and absorb in their work with high levels of intrinsic motivation and enjoyment. Ultimately, such employees may tend to experience the flow at work. Formerly, research related to AC mainly concentrated on the relationship between commitment and well-being. Researchers posited that employees with high levels of commitment experience less stress-related factors at the workplace or have better access to such resources which provide support to deal with stresses and therefore, such employees are more likely to enhance their well-being (Meyer and Maltin, 2010). The results of an empirical study have also confirmed that AC has a positive relationship with psychological well-being (Rivkin et al., 2018). The present paper is in line with these studies as it also highlights that AC has a positive relationship with the flow (i.e., experiential well-being) (Ilies et al., 2016). Third, we have proposed that a high level of EI is expected to increase the strength of the relationships among HIHRM practices, AC, and work-related flow. The motivation mechanism in our research model relied on the workers’ ability to understand and interpret the positive emotional information received from the implementation of HIHRM practices, and to manage and use this emotional detail to facilitate their work attitude and behavior. It implies that employees with high levels of EI are better able to sense positive signals while neglecting any negative signals and facilitate themselves to be intrinsically motivated and absorbed in their work with enjoyment. This assumption is consistent with a study by Lan et al. (2017) which confirms that employees’ competence to comprehend the emotional information and emotional support received from their supervisors facilitates their performance, and the complete motivational mechanism during this process relies on such an ability of employees.

### Practical Implications

Organizations may succeed to recognize the excellent potential of their workers through the use of the most relevant reward strategies (Cacioppe, 1999). We consider HIHRM practices (i.e., recognition, empowerment, information sharing, fair rewards, and competence development) as the most influential job resources for motivating employees to enhance and full use of their skills and abilities. In a nutshell, organizations particularly focusing on the employees’ well-being may develop an efficacious HRM package by using these HIHRM practices. The recent studies also support this assumption by presenting different positive outcomes of a bundle of these HIHRM practices like work engagement which further predicts learning goal orientation, employees’ trust in the organization that helps them in technology adaptation, and person-organization fit that is further negatively related to burnout (Maden, 2015; Kilroy et al., 2017; Rubel et al., 2017). There is, however, a limitation as HIHRM practices may also be seen as job demands that may lead to job stress mainly when organizations may use these practices to increase employee performance. This implication is particularly important because high job stress may predict more harmful health-related effects like several stress-related diseases and frequent visits to doctors (Sidhu et al., 2020). However, organizations may mitigate such effects by providing more support and control to employees and implementing health promotion and stress management programs (Richardson and Rothstein, 2008; Jensen et al., 2013). Even so, organizations should use HRM practices by taking employees’ psychological, physical, and social well-being into consideration as it can be mutually beneficial in terms of employees overall health, increased performance and reduced organizational costs (Guest, 2017). Organizations should act efficiently and proactively to avoid any future employees’ stress-related issues by implementing HRM practices with idiosyncratic deals (i.e., negotiation of individual HRM practices). An idiosyncratic deal is a customized contract between employees and employers that is mutually beneficial and aligns HRM practices with the main concerns and priorities of employees. Studies in this regard confirm that such deals positively influence eudaimonic well-being that may further foster creative performance (Villajos et al., 2019). The importance of EI has also been identified in this paper. The moderating effect of EI suggests that the HIHRM practices-AC-flow process is cognitive and emotive. So, organizations may make EI as one of the essential employee selection criteria or EI training is suggested. In short, it can be summarized that organizations may actively manage their employees and lead them to work-related flow by implementing a strong HRM system that focuses on increasing employees’ well-being and EI. Organizations may eventually achieve all the benefits which are linked with the flow.

This paper is likely to assist executives, human resource development experts, organizations, and other stakeholders looking for a set of specific practices that may lead to a workforce who may deeply involve in their work, enjoy it with high intrinsic motivation, and perform at an optimum level. It delivers a theoretical basis for future empirical studies on the influence of HIHRM and similar practices on flow experience. However, we expect that flow experience being a total immersion in the activity may make the employees so intensely involved in the activity that they may forget about other important parts of their jobs and lives. Probably, such people may suffer work-life balance and may also experience ample negative effects like addiction and increased risk-taking behavior for having been in a flow state for too long. Taking into consideration the dark side of flow (Partington et al., 2009; Schüler and Nakamura, 2013), organizations should focus to manage flow properly. As the flow model depends on the challenges-skills balance, it implies that managers need to closely assess employees’ skills and challenges. If managers feel that employees have more skills with fewer challenges (i.e., demonstrating boredom), they may continually address it and convert such boredom into flow by giving employees new challenges. On the other hand, if employees are facing anxiety (i.e., have more challenges with fewer skills), managers may either provide them more support and opportunities to increase their skills to overcome the anxiety or may reduce their challenges to facilitate them to experience the flow. Importantly, if managers find that employees stay in a flow state for too long (i.e., having negative effects), managers may simply focus on diverting employees’ attention from that specific task. For example, a skilled game developer may also be assigned the task of web development or software development.

### Limitations and Suggestions for Future Research

We have outlined only recognition, empowerment, information sharing, fair rewards, and competence development practices in our paper to promote the work-related flow. Studies have also reported other HRM practices like work structuring which includes teamwork and cooperative work design, job security, family friendly work practices, and flexible job assignments, etc. While these practices are not the core HRM practices, these are also mentioned as HIHRM practices by some of the researchers (e.g., Camps and Luna-Arocas, 2009; Shih et al., 2010; Searle et al., 2011). Thus, the role and effect of these practices on work-related flow may also be investigated.

The nature and role of leadership concerning the implementation of HIHRM practices is missing in our paper. The implementation of HRM practices in organizations is a critical step and requires such leadership behaviors that may carefully and competently manage it (Vasilaki et al., 2016). Organizations may fail to achieve employee satisfaction, productivity, and desired results if HRM practices are not accurately implemented (Guest, 2002; Fey et al., 2009). Positive leaders may implement HRM practices with a precise focus on employees’ well-being. It is because positive leadership behavior positively influences employees’ positive emotions and positively predicts employees’ well-being (Kelloway et al., 2013). Positive leaders enacting the features of psychological capital (i.e., resilience, self-esteem, hope, and optimism) enhance employees’ positivity and performance (Avey et al., 2011). Moreover, the paper solely focuses on employees’ EI for its impact on HIHRM practices-AC-work-related flow process relationship; however, emotionally intelligent leaders also play a significant role in it. Leaders with high EI levels have a skill set of self-awareness, self-management, relationship management, and social awareness. Therefore, they may excel in relationship management by practicing social awareness and may create an environment of respect and trust and particularly may influence positive attitude and behavior among employees (Landry, 2019). Thus, we expect that management training based on the values of positive leadership while enhancing their EI may help leaders to implement HIHRM practices in effective manners and create such an environment that may help in strengthening the relationships among HIHRM practices, AC, and work-related flow.

The five-factor model of personality is expected to play a significant role in our proposed framework. Individuals with openness to experience trait are curious, creative, imaginative, original, intelligent, broad-minded, unconventional, and eager to learn new things (Barrick et al., 2001; Duff et al., 2004). Individuals with extraversion personality trait are energetic, dynamic, voluble, and may also be intrusive. They have the capability to completely involve in their work. They are excitement-seeking, experience emotions with an optimistic approach, and generally feel happiness. Individuals high on conscientiousness trait enjoy their work and comply with the agreed rules of the organization. They are dependable and planful (Barrick et al., 2001; Vorkapić and Gović, 2016). Agreeableness is based on “cooperation, trustfulness, compliance, and affability” and finally neuroticism (low emotional stability) refers to lack of depression, aggression, anxiety, and personal instability (Barrick et al., 2001). It is expected that employees with such characteristics can be susceptible to enjoy and get the benefit out of core HRM practices like recognition, empowerment, information sharing, fair rewards, and competence development as these practices satisfy the basic psychological needs by enhancing motivation, providing autonomy, and increasing skills, knowledge, and abilities. Further, a meta-analytic examination has revealed that all these personality traits may positively affect AC (Choi et al., 2015). So, theoretically, it makes sense that employees having any of these traits may strengthen the positive relationship between HIHRM practices and AC. However, we expect that the investigation of the role of the five-factor model of personality between AC and flow may produce different results as studies have found that extraversion, conscientiousness, and openness to experience traits are positively related whereas neuroticism (low emotional stability) and agreeableness are negatively related to the facets of flow experience (Ross and Keiser, 2014). Other traits beyond the five-factor model of personality may also matter, such as self-efficacy. Self-efficacy is a belief in one’s own capabilities to complete a task. Studies provide evidence that HRM practices positively enhance self-efficacy that further stimulates higher performance (Tabiu, 2016). Notably, Rodriguez-Sánchez et al. (2011) added self-efficacy in the channel model of flow and expanded it. They explain that a high level of self-efficacy may lead to high levels of challenges and skills and ultimately the more flow. It indicates that the role of self-efficacy as a mediator between HIHRM practices and flow experience is possible and should be explored. Future studies may take the aforementioned key aspects into consideration for the development of new research models.

### Concluding Remarks

The core of HRM practices is to bring out the best in employees. This paper identifies such HIHRM practices which are expected to influence the brain’s highest productive state (i.e., work-related flow). Finally, this paper is helpful and encouraging for those practitioners, researchers, and academicians who want to get insight and interested in conducting empirical studies on these concepts.

## Data Availability Statement

The original contributions presented in the study are included in the article/supplementary material, further inquiries can be directed to the corresponding author/s.

## Author Contributions

All authors listed have made a substantial, direct and intellectual contribution to the work, and approved it for publication.

## Conflict of Interest

The authors declare that the research was conducted in the absence of any commercial or financial relationships that could be construed as a potential conflict of interest.
